# Unveiling Stem Cell Kinetics: Prime Time for Integrating Experimental and Computational Models

**DOI:** 10.3389/fonc.2013.00291

**Published:** 2013-11-28

**Authors:** Heiko Enderling

**Affiliations:** ^1^H. Lee Moffitt Cancer Center & Research Institute, Tampa, FL, USA

**Keywords:** stem cells, integrated computational oncology, agent-based models, radiation, cancer risk

*In vitro* and *in vivo* experiments including stem cells or cancer stem cells are performed at an increasing rate, producing a wealth of exciting and sometimes unexpected, even puzzling data. Intuitive speculation about underlying biological mechanisms often prevails as experimental fishing expeditions are costly and time consuming. Based on first principles of cell kinetics without assumptions about the emerging population dynamics, quantitative mathematical and computational models are emerging as invaluable tools to correlate and rank the likelihood of cell-level hypotheses with population level endpoints. To fully utilize the power of quantitative models, experimental and modeling approaches must be integrated and iteratively inform and validate each other.

I read with great interest the article by Tang et al. (1) and praise their iterative interdisciplinary approach to correlate mammary stem cell kinetics with the elevated breast cancer risk after exposure to irradiation in young girls but not adult women. Experiments performed by Tang et al. show that ionizing irradiation of pubertal mammary glands yields an increase in mammary stem cells, prompting questions about the underlying mechanisms. It has been previously observed that irradiation increases the ratio of stem cells in a population, which has been attributed to better stem cell DNA damage repair mechanisms (2–4). A recent computational model was able to show that decreased radiosensitivity alone is insufficient to achieve the observed increase in stem cell ratio, and that a shift to increased symmetric stem cell division must occur especially during fractionated exposure to irradiation (5). By considering pre- and post-pubertal breast morphologies, Tang et al. use agent-based models (ABMs) to simulate radiation responses of juvenile and adult populations. Surprisingly, radiation-induced cell death did not contribute to increased stem cell frequency independently of the dose delivered. Instead the model revealed that the combination of increased self-renewal and cell proliferation in pubertal mammary glands led to stem cell enrichment. In contrast epithelial-mesenchymal transition (EMT) was shown to increase stem cell frequency not only in pubertal mammary glands but also in adult glands. This latter prediction, however, contradicted *in vivo* data thereby suggesting self-renewal as the primary mechanism behind pubertal stem cell increase. To better evaluate this mechanism, Tang et al. conducted *in vitro* studies on human breast cells and confirmed that cells must extensively proliferate to observe a self-renewal dependent increases in stem and progenitor cell numbers. Taken together, the iterative integration of ABM and *in vitro*/*in vivo* experiments revealed that single-cell kinetics and population level dynamics of mammary duct development render pubertal women susceptible to radiation-induced increase in mammary stem cells, which significantly increases the risk of developing aggressive breast cancer later in life (6).

The predictive power of quantitative models lies in the biologically informed formalization of interacting mechanisms. The rates at which these mechanisms occur, however, are encoded in model parameters, demanding a quest for parameter combinations that best match experimental data. The simple model by Tang et al. has 10 parameters. If for each parameter nine different values are considered, then the combination of all possible parameters yields one billion different versions of a stochastic model for a single experimental condition. Exploration of this parameter space is experimentally unfeasible but computationally tractable. ABMs can be subjected to brute force parameter sweeps or genetic algorithms on supercomputer clusters to identify parameters and thus mechanisms that are essential and mechanisms that are unlikely to contribute to specific observations. These invaluable insights into systems dynamics enable targeted biological experimentation and validation.

While Tang et al. excellently dissect individual mechanisms that may underlie pubertal stem cell enrichment after irradiation, combinations of different mechanisms and their additive or potentially synergistic responses are neglected. The low doses of irradiation do not induce significant cell kill or cytostasis; rather they appear to initiate a milieu of activated cytokines and growth factors that prompt a transient local and systemic response permissive of stem cell activation comparable to inflammation or wound healing (7). While upregulation of symmetric division and stem cell renewal may remain the pivotal response, other mechanisms may not have to be outright rejected but could contribute to an orchestrated systemic response that synergistically enhances stem cell activity after non-physiologic perturbation. Understanding radiation-induced activation of the cellular microenvironment, and stem cell niches in particular, offers novel opportunities for counteracting stem cell enrichment after pubertal exposure to irradiation. Furthering the successful application of quantitative models, plausible countermeasures and their combined action can be simulated and system responses predicted, which will guide extensive and expensive *in vitro* and *in vivo* experimentation and accelerate the development toward clinical trials.

Integration of experimental and computational approaches is echoed in numerous integrated departments that arise throughout the academic and industry landscape, as well as federal funding programs that require the marriage of quantitative and experimental sciences to synergistically advance the life sciences. Stem cell biology, a rapidly growing field with tremendous attention and promises including novel cancer care, will greatly benefit from the inclusion of mathematics, engineering, physics, and computer science. An integrated dialog and cross-education should be encouraged if not mandated to fully unveil stem cell kinetics.
